# Surface Structure and Grain Boundary Effects on the Oxygen Evolution Reaction at Gold Electrodes

**DOI:** 10.1021/acselectrochem.5c00224

**Published:** 2025-07-15

**Authors:** Xiangdong Xu, Minkyung Kang, Sabrina Yan, Enrico Daviddi, Geoff West, Dimitrios Valavanis, Oluwasegun J. Wahab, Patrick R. Unwin

**Affiliations:** † Department of Chemistry, 2707 University of Warwick, Coventry CV4 7AL, United Kingdom; ‡ School of Chemistry, 4334 The University of Sydney, Camperdown 2006, NSW, Australia; § Warwick Manufacturing Group, 2707 University of Warwick, Coventry CV4 7AL, United Kingdom

**Keywords:** electrocatalysis, oxygen evolution reaction, noble metals, scanning electrochemical cell microscopy (SECCM)

## Abstract

The surface structure of an electrocatalyst plays a crucial role in determining the activity. As a model system, gold has been widely investigated as an electro-oxidation catalyst, although there has been much less research on the oxygen evolution reaction (OER) in the potential region of gold oxidation. Here, we combine voltammetric scanning electrochemical cell microscopy (SECCM) and electron backscatter diffraction (EBSD), at different spatial and angular resolutions, respectively, to correlate the local crystallographic structure of polycrystalline gold—focusing on grains close to (113), (011), (114), and (111) orientations—with the electrocatalytic behavior for the OER. By recording potential-dependent electrochemical movies, grain-dependent gold oxidation and gold oxide reduction are clearly visualized. In contrast, the OER demonstrates less grain dependence. High-resolution SECCM also provides a powerful way to map the electrochemical activity for OER at grain boundaries, where distinct boundaries exhibit either enhanced or suppressed activity, revealing a strong correlation with the electrochemical active surface area, together with effects from surface roughness and dislocation densities. This latter aspect of the work further emphasizes the important role of grain boundaries in electrocatalysis and the ability of SECCM to directly target and map the activity of these underexplored regions.

## Graphical abstract

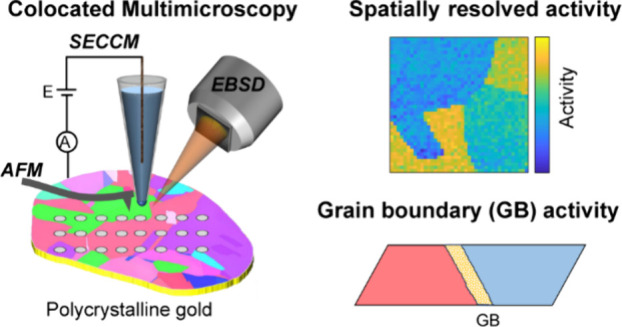

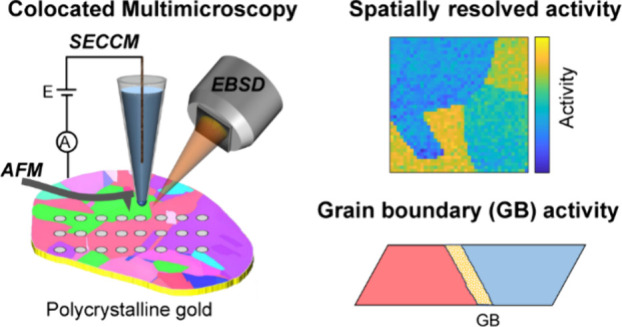

## Introduction

Electrochemical water splitting involves two half-cell reactions that are being intensely investigated: the hydrogen evolution reaction (HER) and the oxygen evolution reaction (OER). The practical and scalable implementation of water splitting electrolysis presently faces challenges because the kinetics of the OER process, in particular, is sluggish. While earth-abundant transition-metal-based catalysts are being studied intensively for OER,^1–3^ noble-metal-based nanostructured materials remain of interest.^4–10^

Gold has been widely investigated as an electrode material for a variety of electrooxidation processes, including the OER. The electrochemistry of gold electrodes in aqueous solution involves a sequence of inter-related potential-dependent processes. Although it has been argued that molecular water adsorbs on the gold electrode surface at low anodic potentials,^11,12^ it is generally accepted that anions in the electrolyte first cover the gold surface in this potential region.^13,14^ In the potential range from ca. 1.1 to 1.8 V vs. RHE, surface anions are replaced by O or OH species, which ultimately reach monolayer coverage.^13^ At more positive potentials, above ca. 1.8 V, site exchange between these oxygen-containing species and gold atoms occurs, leading to the formation of gold oxyhydroxide, AuOOH, with a formal oxidation state of +3.^15–18^ The OER mechanism itself is also under debate. The overall process is AuOOH → Au + O_2_ + H^+^ + *e* ^–^,^11,19,20^ but it has been proposed that oxygen initially evolves from surface oxide via a disproportionation reaction, then starts to evolve from electrolyte via an “oxygen exchange mechanism”.^18,21–23^

The gold electrooxidation process is structure-dependent,^13,24–26^ but the relevance of surface structure for the OER process is unclear. From the results of second harmonic imaging studies,^27^ it was suggested that less than 1% of the surface area of a polycrystalline gold electrode, consisting of two different active sites, contributed to the OER activity of the whole electrode, as determined by visualizing oxygen bubble evolution, which was found to occur at discrete sites.^15^ To explore and clarify the effect of grain structure and grain boundaries (i.e., crystallographic properties) in the OER reaction, we employ scanning electrochemical cell microscopy (SECCM) in tandem with colocated electron backscatter diffraction (EBSD), in order to better understand the role of gold structure on the OER reaction. We prepared a polycrystalline gold nugget as the working electrode (electrocatalyst) to conduct both low-resolution and high-resolution (HR)-SECCM measurement in order to study grain and grain boundary effects, respectively. The combination of SECCM and EBSD is becoming a powerful and straightforward way to make a clear correlation between structure (post-activity analysis) and activity.^28–33^

## Experimental Section

### Preparation of Gold Nugget

A gold wire (0.25 mm in diameter, 99.99+%, Goodfellow, UK) was melted with a butane torch, producing a sphere at the end of the wire, which was quickly cooled in deionized water (18.2 MΩ.cm at 25°C, ELGA Labwater). This process was repeated at least five times resulting in a gold ball of 1-2 mm diameter at one end of the wire. The gold ball was compressed between two silicon wafers (Inseto, UK) using a toolmaker’s vice, to produce a gold nugget. Note that the wafers were cleaned with acetone, isopropanol and deionized water and dried with nitrogen gas before compressing the gold ball. The gold nugget was then annealed with the butane torch. Finally, the flattened surface of interest in the gold nugget was cleaned with broad ion beam milling (Hitachi IM4000Plus, Japan) and then used directly for SECCM measurements.

### Pipette, Quasi-Reference Electrodes, and Electrolyte

Single-barrel nanopipettes (see the Supporting Information S1 for detailed pulling parameters) were fabricated using a P2000 laser-based micropipette puller (Sutter Instruments, US) from capillary tubes supplied by Harvard Apparatus: 350 nm diameter nanopipettes from borosilicate (1.2 OD × 0.69 ID × 100 mm, GC120F-10); and 30 nm nanopipettes from quartz (1.0 OD × 0.50 ID × 100 mm, QF100-50-10, Sutter Instrument). AgCl-coated Ag (Ag/AgCl) quasi reference counter electrodes (QRCEs) were prepared from Ag wire (0.125 mm in diameter, 99.99+%, Goodfellow, UK) by oxidation with voltages ranging between +5 to +7 V vs. Pt wire in a saturated KCl (99%, Sigma-Aldrich) solution for 10-15 mins. Long-term stability of Ag/AgCl QRCEs has been shown in a previous work,^34^ and the QRCEs were routinely calibrated against a commercial leakless Ag/AgCl reference electrode (3.4 M KCl, LF-2-100, Innovative Instruments, Inc., US) both before and immediately after each SECCM experiment.^34^ The freshly prepared 0.5 M H_2_SO_4_ (99.999%, Sigma-Aldrich) solutions were used for both macroscale and SECCM experiments.

### SECCM Measurements

A home-built multifunctional SECCM instrument (Figure 1a), described in detail elsewhere,^30,35–37^ was used. Briefly, the SECCM instrument was placed in a Faraday cage, equipped with heat sinks and acoustic foam, in order to minimize electrical and acoustic noise, as well as thermal drift. The Faraday cage was placed on an optical tabletop (RS 2000, Newport) to minimize mechanical vibration. Data acquisition and instrument control relied on an FPGA card (PCIe-7852R, National Instruments) which was controlled by Labview 2019 software, running the Warwick electrochemical scanning probe microscopy (WECSPM, www.warwick.ac.uk/electrochemistry) platform. The current was measured every 4 μs, with 256 points averaged (and one point for data transfer), to give a data acquisition rate of 1028 μs per data point.

**Figure 1:**
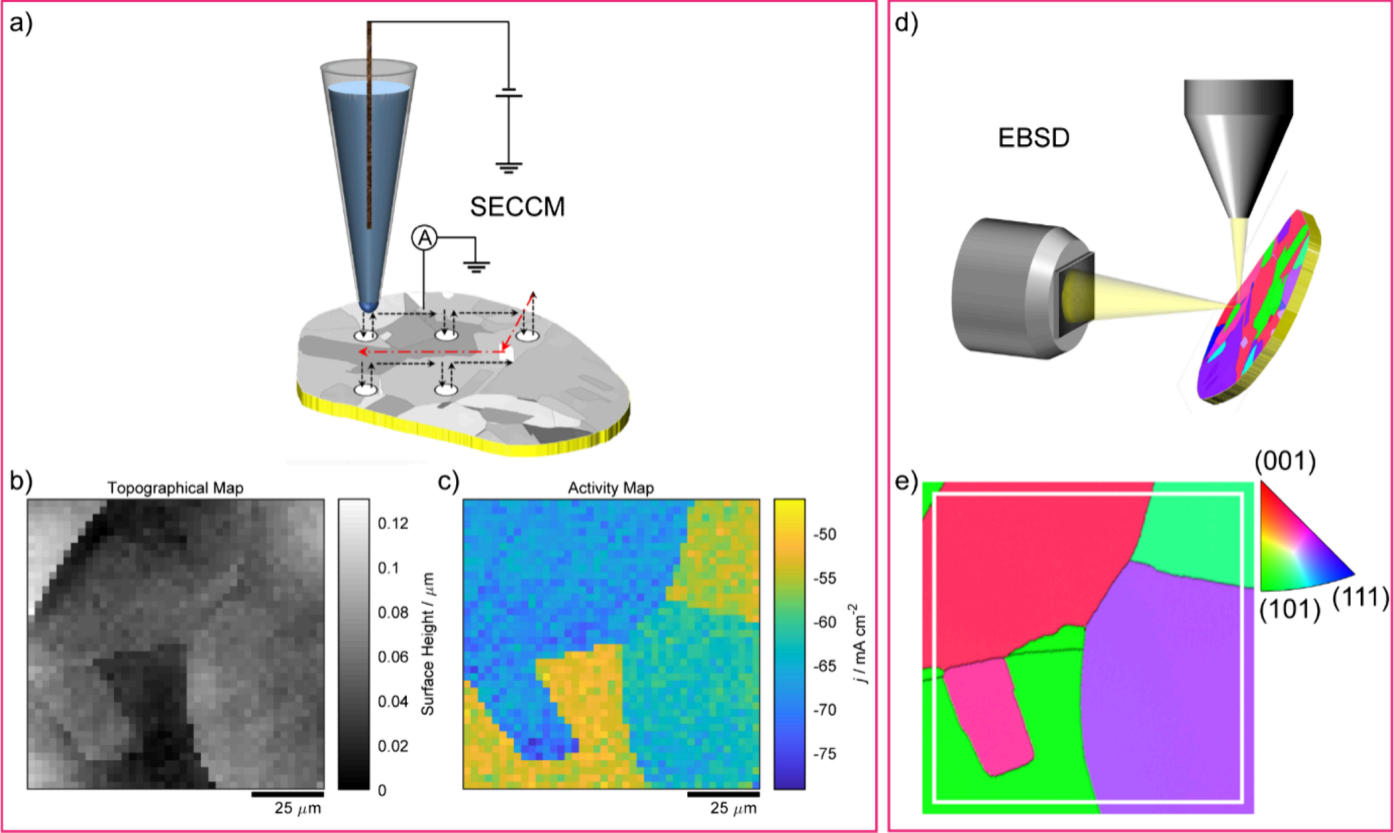
Schematic for correlating electrochemical activity (intrinsic gold redox reaction and OER process) with microstructure of polycrystalline gold surface. (a) SECCM configuration deployed in a hopping mode on the surface of a gold nugget. The single-barrel nanopipette (350 nm diameter for low-resolution SECCM and 30 nm diameter for HR-SECCM) was filled with 0.5 M H_2_SO_4_ solution and cyclic voltammetry was performed at every point of contact of the nanopipette meniscus with the surface. The arrows indicate the movement of SECCM probe across the surface of the gold working electrode. (b) Typical illustrative topography and (c) activity map from an SECCM scan (*vide intra*). (d) Schematic of colocated electron backscatter diffraction (EBSD) measurement on the polycrystalline gold surface and (e) the corresponding EBSD map after an SECCM scan (b and c).

The polycrystalline gold nugget that served as the working electrode was placed on a sample holder on top of an *xy*-piezoelectric positioning stage (P-733.2, Physik Instrumente, Germany). The nanopipette, filled with 0.5 M H_2_SO_4_ and equipped with an Ag/AgCl QRCE, was mounted on a z-piezoelectric positioner (P-753.2, Physik Instrumente, Germany). The nanopipette was positioned near the gold surface using micro-positioners (M-461-XYZ-M, Newport, US), which provided coarse movement in x, y, and z axes, guided by optical visualization using an optical camera (PL-B776U camera, 4× lens, Pixelink).

SECCM was operated in hopping mode,^38^ with cyclic voltammetry executed at each location (pixel), where the nanopipette made meniscus contact with the gold surface. The current in the electrochemical cell (formed by the liquid meniscus on the nanopipette and the gold substrate) was measured synchronously throughout the nanopipette movement and applied voltammetric waveform. The initial potential of the gold substrate (working electrode; WE) was set to 0.2 V with respect to Ag/AgCl QRCE. Upon meniscus contact (detected as a sharp increase in the measured current surpassing a threshold value), the vertical motion of the nanopipette was stopped immediately. A triangular potential waveform was applied to the WE from 0.2 to 1.75 V, and then back to −0.2 V with respect to Ag/AgCl QRCE at a scan rate of 2 V ^−1^, after which the nanopipette was retracted by 3 μm at a speed of 3 μm s^−1^ and moved to the next location (pixel) to repeat the same procedure. A relatively high scan rate was possible because of the nanoscale nature of the measurement and was essential to allow imaging on a reasonable time scale. It was also useful in amplifying surface-confined processes. The hopping distance between pixels was 2.5 μm for low-resolution imaging and 80 nm for high-resolution imaging. An example of one completed measurement, showing the lateral movement of the substrate in the *x*-direction, vertical movement of the nanopipette (*z*-direction) and the cyclic voltammetric measurement (current-potential curve) at one location is illustrated in Figure S1 in Supporting Information. The results at each location can be extracted to plot topography (Figure 1b) and activity maps at a particular potential (Figure 1c), to visualize surface topography and the corresponding local electrochemical activity. All the potentials mentioned in this chapter are converted and referred to the reversible hydrogen electrode (RHE), unless otherwise stated.

### Complementary Physical Characterization of the Gold Surface

The SECCM footprints left after the experiments were imaged by field emission scanning electron microscopy (FE-SEM, ZEISS Gemini, Germany) and their geometric areas used to estimate current densities. Sample topography was characterized by atomic force microscopy (AFM, Bruker Innova, Germany), to evaluate the surface roughness and surface area at the nanoscale. EBSD (Figure 1d) was used to study the grain crystallographic orientations (Figure 1e) of the same area where the SECCM/AFM imaging was done. High angular resolution (HR)-EBSD analysis was also carried out to investigate the stress/strain level across grain boundaries. A JEOL 7800F with a symmetry EBSD (Oxford Instruments, UK) detector was used in this study. EBSD data were analyzed using AztecCrystal and CrossCourt 4 software.

## Results and Discussion

### Cyclic Voltammetry on Gold: Macroscale and Nanoscale

To determine the potential window of the electrochemical processes of interest, macroscale voltammetric measurements (CompactStat.h, Ivium Technologies, Netherlands) were first performed on a polycrystalline gold ball working electrode (geometric area ca. 0.015 cm^2^) in 0.5 M H_2_SO_4_. A three-electrode cell consisting of a glassy carbon rod and commercial Ag/AgCl (3.4 M KCl) as counter and reference electrodes, respectively, was used. A representative cyclic voltammogram (CV), where the applied potential was scanned anodically in the forward sweep, from 0.58 V vs. RHE at a scan rate of 50 mV s^–1^ is shown in Figure 2a. An oxidation process starts at approximately 1.36 V vs. RHE, and is sustained until the reverse scan at 2.36 V vs. RHE. The first relatively sharp oxidation process, with a peak potential at 1.42 V vs. RHE, can be attributed to the deposition of OH, which is accompanied by desorption of other electrolyte anions.^13^ The second oxidation process, with a peak potential of 1.63 V vs. RHE, can be attributed to exchange between the absorbed oxide species with surface gold atoms, which is commonly known as the “replacement-turnover” process.^13,25^ The broad plateau between these two peaks is due to complex oxidation processes (e.g., deposition of OH species in the sublattice).^13,39^

**Figure 2:**
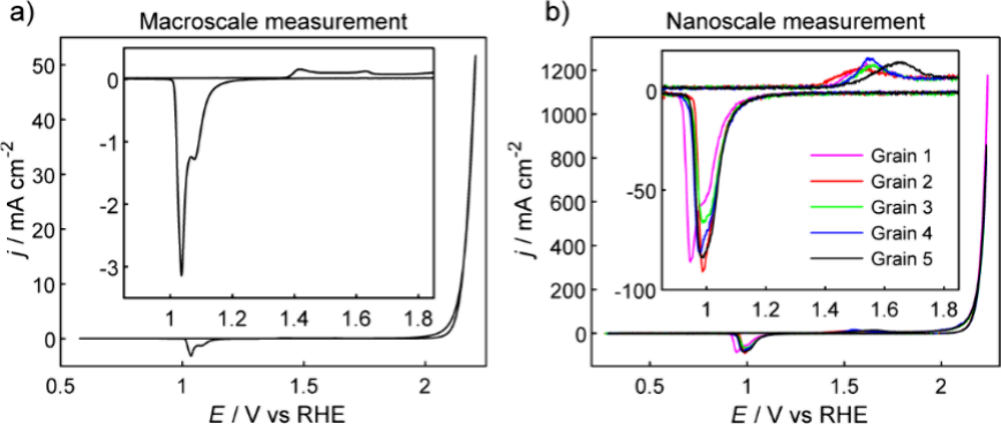
Cyclic voltammograms obtained at the (a) macroscale using a polycrystalline gold nugget as a working electrode in the standard three-electrode configuration (scan rate: 50 mV s^–1^) and (b) nanoscale using a polycrystalline gold nugget as the working electrode in the SECCM format with a 350 nm diameter nanopipette (scan rate: 2 V s^–1^). The inset images are magnified cyclic voltammograms in the potential window of 0.85–1.85 V.

The two reduction peaks, located at 1.08 and 1.04 V vs. RHE, are due to the reduction of gold surface oxides, accompanied by the re-adsorption of electrolyte anions.^40^ Generally, only one or two reduction peaks are visible in acid solution, assigned to the reduction of β-oxide and α-oxide.^39,41^ The reduction peak positions depend on several variables, e.g., potential range of the CV measurement, scan rate, number of cycles, thickness of the surface oxide layer, nature of electrolytes, etc.^42–44^ Variations in peak positions are observed from the macroscale voltammetry measurements when using different potential ranges and scan rates (Supporting Information, Figure S2). The growth of the gold oxide layer, as indicated by the reduction charges (Supporting Information, Table S1), exhibited comparable behavior across various scan rates. This consistency validates the use of a scan rate of 2 V s^–1^ as a reasonable choice for SECCM measurements.

Representative CVs from five different grains (Grain 1-5), acquired on the polycrystalline gold nugget through nanoscale SECCM measurements, are shown in Figure 2b. With the flame-annealing method, the grain sizes of gold range from 10s to 100s μm (*vide infra*). Given that the probe diameter is 350 nm, the individual measurements were conducted within single grains. The profiles of the nanoscale CVs are comparable to the macroscale measurements, with surface oxidation beginning at approximately 1.36 V vs. RHE. The gold oxidation peaks occur in the range from 1.50 to 1.65 V vs. RHE, and the reduction of gold oxides occurs between 0.85 and 1.40 V vs. RHE during the reverse scan. The relationship between the voltammetric profiles and surface structure can be examined by coupling SECCM imaging with correlative EBSD analysis, providing a pseudo-single-crystal approach to electrochemical analysis.^29^ This method provides information on crystallographic orientations and grain boundaries, which can significantly impact the electrochemistry of gold surfaces.

### Grain-Dependent Electrochemistry of Gold

For spatially resolved SECCM, voltammetric measurements were executed at a scan rate of 2 V s^−1^, in the potential range from 0.29 to 2.24 V vs. RHE. Two voltammetric cycles were recorded at each location of the scan and the resulting electrochemical behavior was captured as a CV-SECCM movie (Supporting Information, Movie S1) of electrochemical activity (current density, *j*) as a function of potential. The spatially resolved electrochemical movie, compiled from activity maps at every mV, includes 920 independent voltammetric measurements at different locations across the surface of the gold nugget, with a pixel density of 0.16 pixels μm^−2^. The scanned surface is shown in the SEM image (Figure 3a) and EBSD map (Figure 3b). As can be seen in the SEM image (Figure 3a), each individual pixel of the SECCM “footprint”, where the confined liquid meniscus cell contacted the gold surface for each CV measurement, was around 330 nm in diameter. Based on the geometric area of each pixel footprint, estimated from the SEM image, the measured current was converted to current density to present specific frames or voltammograms (Figure 1 and Figure 2).

**Figure 3:**
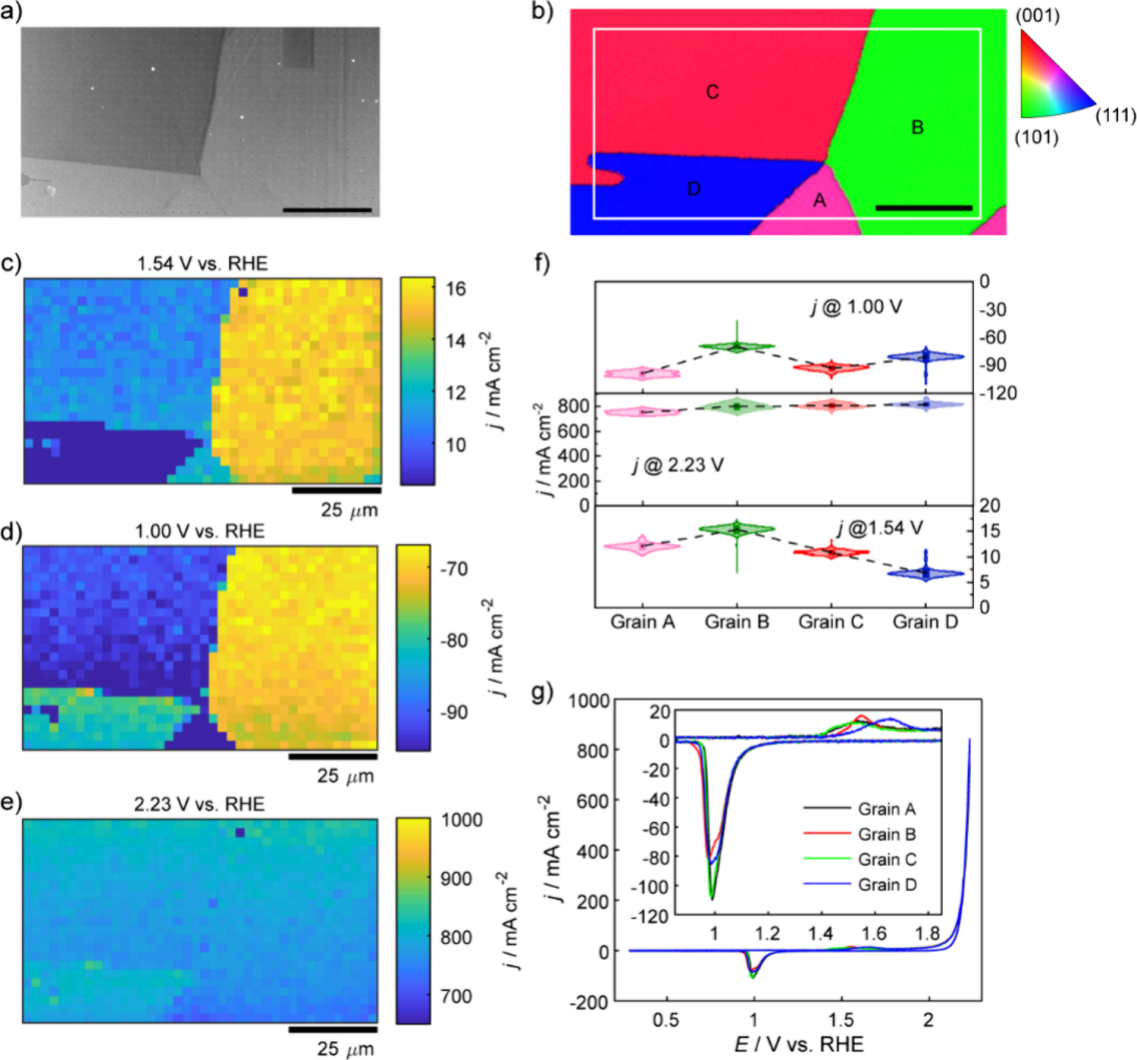
(a) SEM image and (b) corresponding EBSD grain orientation map of the surface of the polycrystalline gold nugget, acquired after the SECCM experiment. The marked white box indicates the scanned areas with SECCM. The scale bars are 25 μm. Spatially resolved equipotential snapshot images from Movie S1 at specific potentials of (c) 1.54, (d) 1.00, and (e) 2.23 V, respectively. (f) Violin plots of current densities of four different grains, two potentials from the anodic scan, (c) 1.54 V and (e) 2.23 V, and one from cathodic scan, (d) 1.00 V. The dashed lines indicate the mean values of current density at specific potentials across different grains. (g) Representative CVs of four grains obtained from the SECCM measurement. The inset images show magnified CVs in the potential window region 0.85–1.85 V.

Spatially resolved equipotential snapshots from Movie S1 presented in Figure 3c–e, represent regions for the gold surface oxidation at 1.54 V, gold oxide reduction at 1.00 V (reverse scan) and OER at 2.23 V, respectively. The correlation of the activity maps with EBSD indicates that gold oxidation (Figure 3c) and gold oxide reduction (Figure 3d) are highly grain-dependent, showing high variation in current density at 1.54 and 1.00 V, respectively, due to the peak shift being determined by their grain orientation. In contrast, OER activity (Figure 3e) is almost independent of grain orientation and the surface is relatively uniformly active at this potential. For detailed grain comparison, the SECCM map was divided into four regions corresponding to different grains, labeled as A, B, C and D (Figure 3b), which are close to the (113), (011), (114), and (111) facets, respectively. The histogram of current densities of each grain at selected potentials is shown in Figure 3f. The current distribution of each grain is found to be rather similar within reasonable standard distributions (Supporting Information, Table S2).

In the gold oxidation region, substantial differences in electrochemical response were observed across individual grains (Figure 3c), revealed by representative nanoscale CVs (Figure 3g). Figure 4 provides further information on the gold oxidation peak potential and charge, offering a more detailed analysis of the electrochemical response across individual grains. The average peak potentials for grains A, B, C, and D were determined to be 1.54 ± 0.01 V, 1.55 ± 0.01 V, 1.52 ± 0.01 V, and 1.65 ± 0.02 V, respectively (Figure 4a) and the corresponding charge within the 1.2–2.13 V region (associated with gold oxidation) is also grain-dependent (Figure 4b). The average transferred charge for grains A, B, C, and D was 3.54 ± 0.05 pC, 3.44 ± 0.04 pC, 3.39 ± 0.04 pC, and 3.30 ± 0.07 pC, respectively. Notably, grain D, Au(111), exhibits the highest overpotential for the oxidation process.

**Figure 4:**
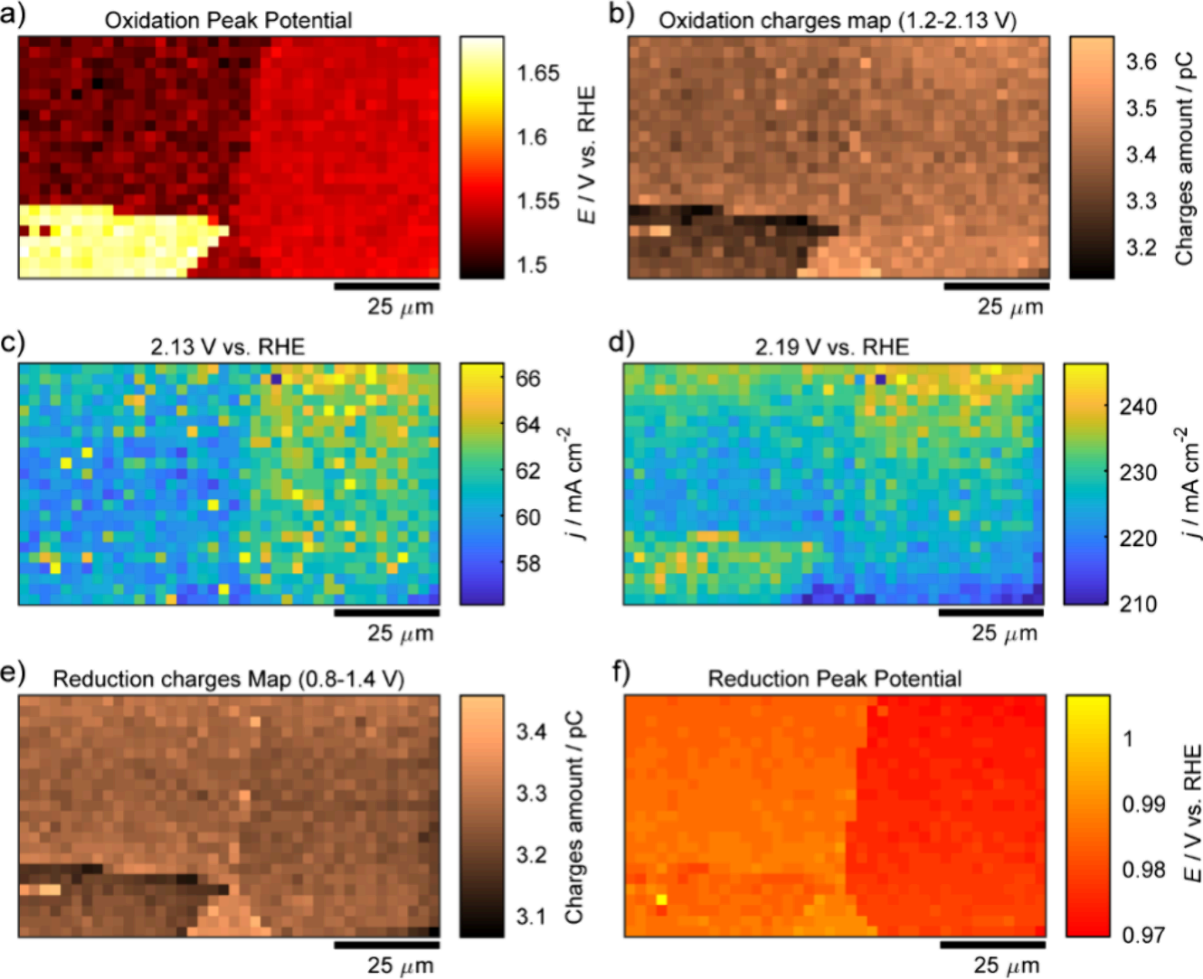
In-depth data analysis based on the SECCM scanning area detailed in Figure 3. (a) The oxidation potential shift map, built by finding the main oxidation peak potential of anodic CV curves of each pixel. (b) The oxidation charges map, built by integrating the anodic CV curves of each pixel in the potential window of 1.20–2.13 V. Spatially resolved equipotential snapshot images from Movie S1 at specific potentials of (c) 2.13 V and (d) 2.19 V. (e) The reduction charges map, built by integrating the cathodic CV curves of each pixel in the potential window of 0.8–1.4 V, which covers all the reduction peak region. (f) The reduction potential shift map, built by finding the main reduction peak potential of cathodic CV curves of each pixel.

The observed grain heterogeneity in the surface oxidation of gold can be ascribed to differences in surface blocking by electrolyte anions, which compete with OH species for adsorption sites during oxidation.^27,45^ The surface symmetry of different grains plays a key role in determining the kinetics of gold oxidation by modulating the extent of anion adsorption and surface blocking. For example, sulfate anions are known to adsorb more strongly on surfaces with trigonal symmetry, which then requires higher overpotentials to initiate surface oxidation.^13^ In this work, grain D exhibits a symmetry closer to that of a trigonal-face, which correlates with the more sluggish oxidation process observed.

Equipotential snapshots in the OER region at 2.13 V (Figure 4c) and 2.19 V (Figure 4d) highlight grain-specific behavior, as visualized in spatially resolved electrochemical maps (Movie S1). The OER activity at 2.19 V exhibits minor variation across the four grains studied (Supporting Information, Table S2), consistent with prior findings on single-crystal gold surfaces. For example, (100), (111), and (110) facets display only slight differences in Tafel slopes, typically ranging from 42 to 46 mV dec^–1^ at potentials above 2 V.^18,46^ Nevertheless, subtle variations can still be observed, which are likely due to the differing affinities of electrolyte anions for adsorption on specific grain surfaces. This variation in anion adsorption hinders the formation of AuO_*x*_, particularly on grain D, consequently affecting OER activity.

Notably, the average current density for grain D increases most significantly between 2.14 and 2.19 V, with rates of 3.60, 3.77, 3.69, and 3.95 mA cm^–2^ mV^–1^ for grains A, B, C, and D (see section S5.2 of the Supporting Information for detailed analysis), respectively. Within this potential range, OER is catalyzed by the gold oxide layer through a disproportionation mechanism (5AuOOH → 2Au_2_O_3_ + Au + 2O_2_ + 5H^+^ + 5e^–^),^18^ which is grain-dependent in both its formation and thickness.^47^ This mechanism entails site exchange between surface oxygen-containing species and bulk gold atoms, necessitating diffusion through the gold oxide layer. Stronger anion adsorption on grain D results in delayed AuO_*x*_ growth, producing a relatively thinner oxide film^48^ and facilitating faster electron transfer compared to other grains. This behavior is supported by the charge map of gold oxide reduction (Figure 4e), which shows a lower total charge for grain D, indicating a thinner oxide layer.^47^

Although the differences in OER activity among grains appear small,^18^ these variations are made more apparent through the large statistics provided by SECCM on multigrain index samples (*vide infra*). OER on gold involves an increasingly oxidized surface, where the thermodynamic driving force from the surface oxide film is greater than that of bulk gold.^15,49^ Simultaneously, gold dissolution occurs as surface-active gold cations are replenished from the bulk via a site-exchange mechanism.^50,51^

When comparing the charge maps of gold oxidation at the potential range from 1.2 to 1.54 V, 1.85 V, 2.0 V, 2.13, 2.19, and 2.23 V (Supporting Information, Figure S4), the charge differences among grains diminish with increasing potential, particularly as the OER proceeds (Supporting Information, Figure S4f and Table S3). At higher potentials the OER is much less grain-dependent overall.

Similar to gold surface oxidation, gold oxide reduction exhibits clear grain-dependent behavior, as shown in both the current density (Figure 3d) and peak potential (Figure 4f) maps. Such variations in reduction peak values and shapes have been previously attributed to several factors, including anodic polarization time, the oxidation upper potential limit (the maximum potential in the CV window), scan rate, chemical heterogeneity of Au oxide species (e.g., Au_2_O_3_, AuOOH) and the thickness of the oxide layer formed.^39,43,51–53^ Further analysis of gold oxide reduction was conducted on representative CVs of individual grains (Figure 3g). Both grains B and D exhibit two distinct reduction peaks—a main peak accompanied by a shoulder—while grain B demonstrates the most negative peak potential. In contrast, grains A and C exhibit higher peak current densities during reduction. This grain-dependent behavior is further reflected in the charge map for gold oxides reduction (Figure 4e), which indicates that the total charge for each grain follows the order: Q_A_ > Q_C_ ≈ Q_B_ > Q_D_, with values of 3.36 ± 0.03 pC, 3.27 ± 0.03 pC, 3.25 ± 0.03 pC, and 3.22 ± 0.05 pC, respectively. This trend implies a variation in gold oxide layer thickness across grains, with grain A having the thickest oxide layer, followed by grains C ≈ B, and D. Furthermore, comparison with macroscale and nanoscale CV curves provided in the Supporting Information (Supporting Information, Figure S2) highlights that the number of reduction peaks is closely related to the extent of the OER or the oxidation upper potential limit.

Eighteen additional grains were analyzed using the same approach; SECCM results are available in the Supporting Information (Movie S2, Movie S3, and Movie S4, Figures S5–S7 and Tables S4–S6), which correspond to the discussions of grain A-D (Figure 3 and Figure 4). It is interesting to note that while all the surfaces investigated demonstrate electrochemical activity for the OER, bubble nucleation as a result of OER occurs at a few discrete sites.^15,27^ Heterogeneous oxygen bubble nucleation involves several inter-related factors,^54^ and in the future it would be interesting to link the observations herein, to bubble nucleation. To that end, a combination of SECCM and optical microscopy^55,56^ to precisely correlate electrochemical activity with bubble nucleation^57,58^ events, could providing deeper insights into the mechanisms governing OER at microscale.

### Grain-Boundary Electrochemistry

SECCM is a powerful technique for visualizing electrochemical activity at, and across, grain boundaries at the surfaces of polycrystalline electrodes.^28,30,49,59^ To focus on grain-boundary effects, we employed smaller SECCM probes (diameter = 30 nm) and a reduced hopping distance (80 nm) to conduct HR-SECCM experiments. These included two scans comprising 378 (Figure 5) and 150 (Figure 6) independent measurements, corresponding to a pixel density of 156.25 pixels μm^‑2.^

**Figure 5:**
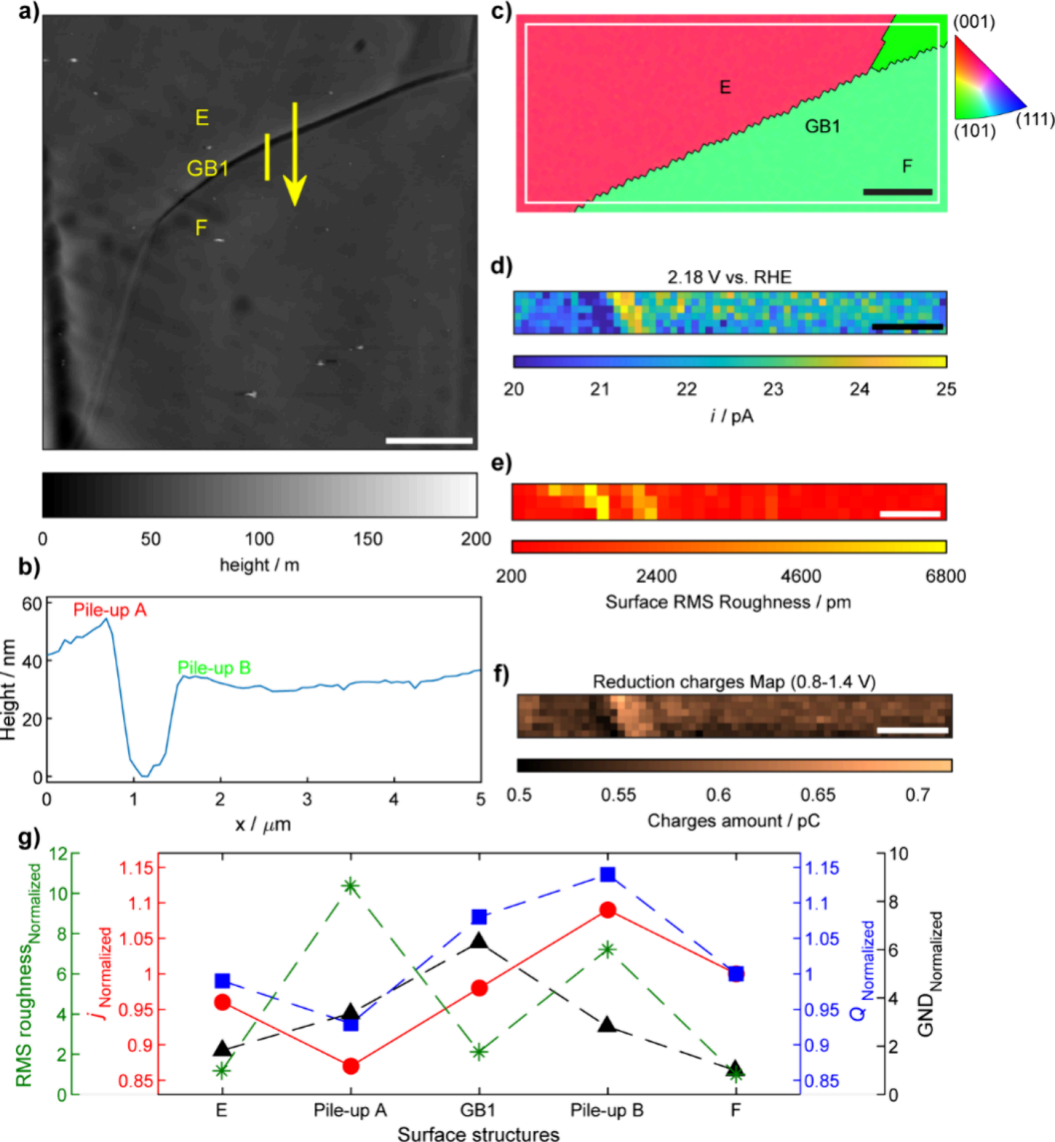
(a) Atomic force microscopy measurement of surface topography in a representative area. The yellow marked line is used for high-resolution (HR)-SECCM mapping, covering two grains (grain E and grain F) and one grain boundary. The scale bar is 7 μm. (b) The line profile of the yellow marked line, along the direction of yellow arrow in a). (c) Corresponding EBSD grain orientation map of the surface of polycrystalline gold nugget, performed after HR-SECCM experiment. Spatially resolved equipotential snapshot images acquired from Movie S5 at a specific potential of (d) 2.18 V. The scale bar is 0.8 μm. (e) The root mean square (RMS) roughness map extracted from (a), with the pixel density of 51 pixels μm^–2^. The scale bar is 0.7 μm. (f) The reduction charges map, built by integrating the cathodic CV curves of each pixel in the potential window of 0.8–1.4 V, which covers all the reduction peak region. The scale bar is 0.8 μm. (g) The plot of the ratio of normalized current density at 2.18 V, RMS roughness, reduction peak charges, and geometrically necessary dislocation (GND) density.

**Figure 6:**
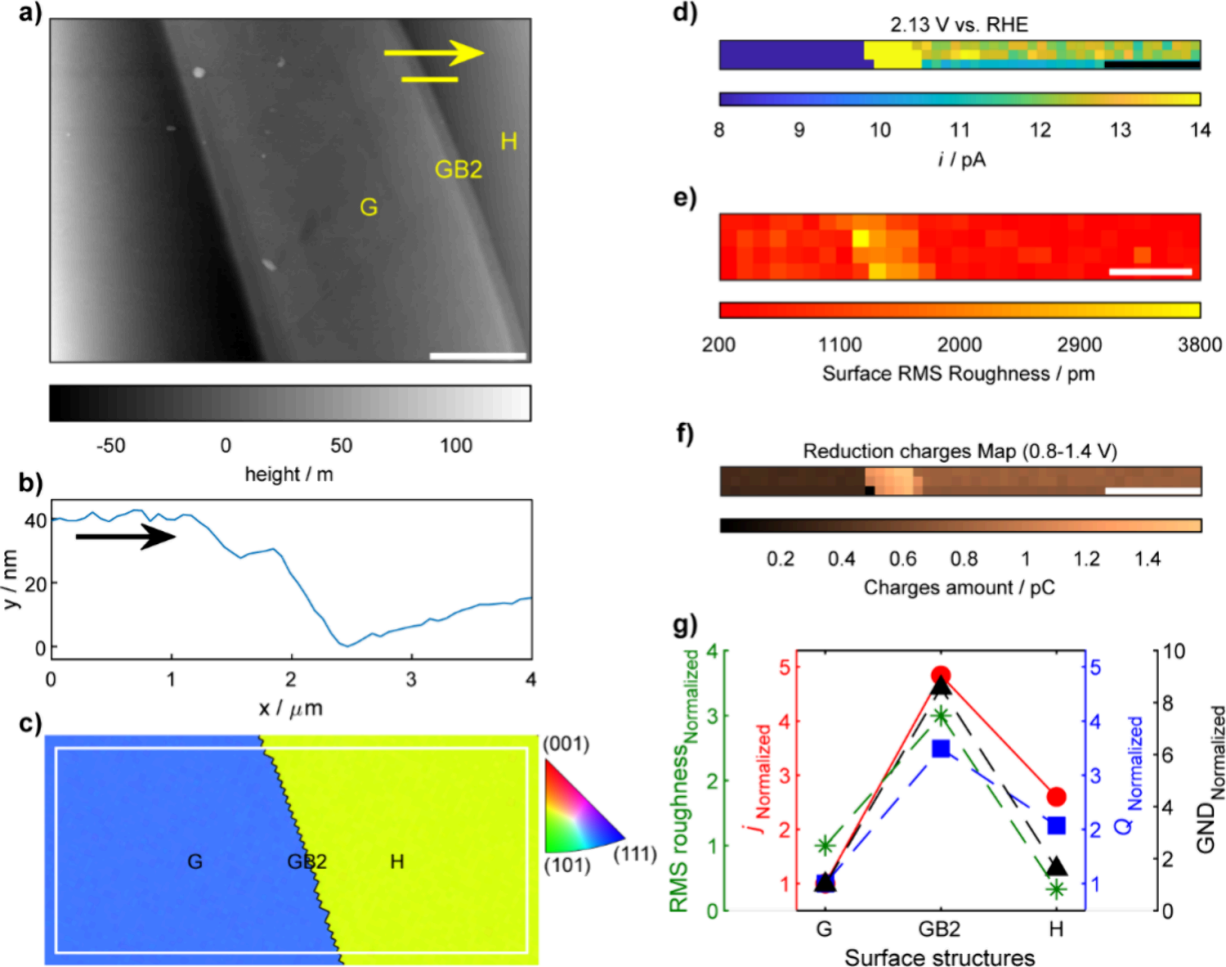
(a) Atomic force microscopy measurement of surface topography in a representative area. The yellow marked line is used for HR-SECCM mapping, covering two grains (grain G and grain H) and one grain boundary (GB2). The scale bar is 7 μm. (b) The line profile of the yellow marked line, along the direction of yellow arrow in a). (c) The corresponding EBSD grain orientation map of the surface of polycrystalline gold nugget, performed after HR-SECCM experiment. Spatially resolved equipotential snapshot images got from Movie S6 at a specific potential of (d) 2.13 V. The scale bar is 0.8 μm. (e) RMS roughness map extracted from (a), with the pixel density of 51 pixels μm^–2^. The scale bar is 0.7 μm. (f) The reduction charges map, built by integrating the cathodic CV curves of each pixel in the potential window of 0.8–1.4 V, which covers all the reduction peak region. The scale bar is 0.8 μm. (g) The plot of the ratio of normalized current density at 2.13 V, RMS roughness, reduction peak charges, and geometrically necessary dislocation (GND) density.

We could not image the footprints with these ultrasmall tips by post-experiment SEM images. To eliminate the possibility of electrowetting at high potentials, we conducted a series of SECCM CV measurements at varying upper potential limits (1.28, 1.63, 1,73, 1.83, 1.93, 2.03, 2.13, and 2.23 V) with a tip diameter of 350 nm, thereby exploring three distinct potential regions: double layer, gold oxidation, and OER. The resulting droplet footprints, confirmed by post-experiment SEM images (a droplet footprint diameter of approximately 500 nm), remained consistent in size across the different upper potentials (Supporting Information, Figure S8). This observation aligns with other SECCM studies, which indicate that small surface features do not significantly affect the droplet stability even at high resolution.^60–62^

To elucidate the relationship between surface structure and grain-boundary electrocatalytic performance, two representative areas, designated as Region I and Region II (Figure 5a and Figure 6a), were selected based on their distinct morphological features. The surface height line profile for Region I (Figure 5b) reveals a V-shaped morphology at the grain boundary (40°, GB1, Figure 5c). Spatially resolved equipotential snapshots at specific potentials (Figure 5d and Figure S9a and b in the Supporting Information) and corresponding CVs (Supporting Information, Figure S9c–e) exhibit electrochemical activity variations across GB1 (e.g., OER at 2.18 V, Figure 5d). The colocated AFM scan across GB1 produced a root mean square (RMS) roughness map (Figure 5e) with a pixel density of 51 pixels μm^–2^. Notably, two regions adjacent to the grain boundary displayed increased RMS roughness, attributed to the formation of two pile-ups (pile-up A and pile-up B, *vide infra*). The RMS roughness ratio of grain E:pile-up A:GB1:pile-up B:grain F was approximately 1.18:10.38:2.12:7.21:1. However, when compared with the OER current ratio at 2.18 V across grain E, pile-up A, GB1, pile-up B, and grain F (0.96:0.87:0.98:1.09:1, Table S7 in the Supporting Information), it was determined that the gold oxide reduction peak charges (Figure 5f) predominantly influences the OER activity (Figure 5g). The influence of reduction peak charges is greater than that of the RMS roughness.

In the second region (Region II), the surface height line profile (Figure 6b) reveals a step-like morphology corresponding to the grain boundary (Σ3 along ⟨111⟩ direction, GB2, Figure 6c). Spatially resolved equipotential snapshots at specific potentials (Figure 6d and Figure S10a and b in the Supporting Information) and corresponding CVs (Supporting Information, Figure S10c) indicate enhanced electrochemical activity (e.g., OER at 2.13 V, Figure 6d) at GB2. The RMS roughness map (Figure 6e) shows that the roughness ratio between grain G, GB2, and grain H is approximately 1:3:0.33. Similarly, when comparing this with the ratio of OER current at 2.13 V across grain G, GB2, and grain H (1:4.84:2.60, Table S8), the reduction peak charges (Figure 6f) can affect the OER activity (Figure 6g). The RMS roughness may also have a comparable effect.

Numerous theoretical and experimental studies have explored the influence of various grain boundary characteristics - such as type,^63,64^ density,^65^ micro-strain effects,^66^ and dislocation^28^ - on catalytic activity. Dislocations, common defects in solid materials, have been extensively studied to understand their relationship with catalytic activity. Depending on the reaction, dislocation density can either enhance or inhibit catalytic performance.^67–70^ In this work, HR-EBSD was utilized to correlate electrochemical activity with local dislocation densities near grain boundaries.

The geometrically necessary dislocation (GND) density, derived from HR-EBSD data, reveals two main types of dislocations: edge dislocations (Supporting Information, Figure S11a and c) and screw dislocations (Supporting Information, Figure S11b and d). It is important to note that the interaction between crystal orientations and grain boundaries introduces complexities that make it challenging to directly correlate dislocation density with catalytic activity across different grains.^71^ For example, in Region I, the GND density ratio for grain E, pile-up A, GB1, pile-up B, and grain F is 1.83:3.36:6.30:2.82:1 (Supporting Information, Figure S11e). Compared with the OER current ratio at 2.18 V (Supporting Information, Table S7), this possibly suggests a promoting effect of GB1 on OER within grain F, but a hindering effect within grain E. In Region II, GB2 exhibits significantly higher OER activity compared to grains G and H, correspondingly, the GND density map (Supporting Information, Figure S11f) provides a ratio of 1:8.56:1.61, indicating that increased dislocation density at the grain boundary likely contributes to enhanced catalytic activity. These findings highlight SECCM’s capability to visualize catalytic activity differences arising from subtle strain and dislocation variations. These new results should inspire further analysis across a broader range of grains and systems to determine whether unusual activity is driven by individual structural effects or the interplay of multiple factors.

## Conclusions

In this study, SECCM was employed to explore the oxidation and reduction behaviors of polycrystalline gold surfaces. By integrating SECCM with EBSD, we successfully established correlations between electrochemical responses and grain orientations. Our findings demonstrate that gold oxidation and reduction are strongly dependent on grain orientation, with significant variations in the peak potential for oxidation and the growth of the oxide layer. Conversely, OER exhibited minimal grain dependence, particularly at higher potentials where oxygen evolves from the bulk solution. As the potential increased, the differences in oxidation charges among grains diminished. Thus, all gold surfaces exhibit closely similar OER activity for the current densities (potential range) that we have explored. Earlier work suggests that bubble nucleation and gas evolution occur at distinct sites,^15,27^ and the relationship between intrinsic activity and bubble nucleation is an aspect of the OER system, that warrants further exploration to fully understand the underlying mechanisms at the micro- and nanoscale.

The study demonstrates that defect-rich grain boundaries exhibit significantly higher catalytic activity compared to the individual grains. The accumulation of defects provides more active sites which is beneficial to improve the OER catalytic performance. Electrochemical analysis of grain boundaries should be still approached from multiple angles, considering factors such as defect density, reduction peak charge, surface roughness, and other local structural features. This study also highlights the inherent complexity in evaluating the role of grain boundaries in electrocatalytic processes, where activity may be governed by individual structural features or the interplay of multiple contributing factors.

These insights into the relationship between surface structure, grain orientation, and electrochemical behavior offer valuable guidance for the design of gold-based electrocatalysts. While efforts in large-scale electrocatalysis often focus on controlling nanoparticle shape to expose specific grain orientations and maximize catalytic activity, the inherent presence of various facets and surface defects introduces challenges that require further attention. Proper understanding requires multiscale investigation, for which high-resolution techniques such as HR-SECCM are particularly well-suited.

## Supplementary Material















## Notes

Tip pulling parameters (Section S1), illustration of one completed SECCM measurement (Section S2), additional macroscale cyclic voltammetry on gold (Section S3), additional analysis of SECCM measurement (Sections S5 and S6), SEM image of footprints after SECCM experiments (Section S7), and additional analysis of HR-SECCM measurement (Section S8) as described in main text (PDF)
Movie S1 of SECCM measurement (AVI)
Movie S2 of SECCM measurement (AVI)
Movie S3 of SECCM measurement (AVI)
Movie S4 of SECCM measurement (AVI)
Movie S5 of SECCM measurement (AVI)
Movie S6 of SECCM measurement (AVI)

## ∥

Université Paris Cité, CNRS, ITODYS, F-75013 Paris, France

## ⊥

Rowland Institute, Harvard University, Cambridge, Massachusetts 02142, United States.
