# Supplementary material for: Surface Structure and Grain Boundary Effects on the Oxygen Evolution Reaction at Gold Electrodes
Source: ACS Electrochem. 2025 Jul 15;1(9):1852–62. doi: 10.1021/acselectrochem.5c00224 (PMC12415841; doi:10.1021/acselectrochem.5c00224)
Supplement: Supplementary file 1 [file ec5c00224_si_001.pdf]

Supporting Information

Surface structure and grain boundary effects on the oxygen evolution reaction at gold electrodes

Xiangdong Xu,[a] Minkyung Kang,[b,*] Sabrina Yan,[c] Enrico Daviddi,[a,d] Geoff West,[c] Dimitrios Valavanis,[a] Oluwasegun J. Wahab,[a,e] Patrick R. Unwin[a,*]

[a]Department of Chemistry, University of Warwick, Coventry, CV4 7AL, UK.

[b]School of Chemistry, The University of Sydney, Camperdown, 2006 NSW, Australia.

[c]Warwick Manufacturing Group, University of Warwick, Coventry, CV4 7AL, UK.

[d]Present address: Université Paris Cité, CNRS, ITODYS, F-75013 Paris, France.

[e]Present address: Rowland Institute, Harvard University, Cambridge, Massachusetts 02142, United States

*Corresponding authors: minkyung.kang@sydney.edu.au, p.r.unwin@warwick.ac.uk

Table of Contents

S1. Tip pulling parameters 3

S2. Illustration of one completed SECCM measurement 4

S3. Additional macroscale cyclic voltammetry on gold 5

S4. Movie captions 6

S5. Additional analysis of SECCM measurement in Fig. 3 6

S6. Additional SECCM measurements on gold surface 9

S7. SEM image of footprints after SECCM experiments 12

S8. Additional analysis of HR-SECCM measurement in Fig. 5 and Fig. 6 13

S1. Tip pulling parameters

For 350 nm pipettes:

Line 1: HEAT 340, FIL 3, VEL 30, DEL 250, PUL ---;

Line 2: HEAT 340, FIL 3, VEL 30, DEL 210, PUL 120.

For 30 nm pipettes:

Line 1: HEAT 750, FIL 4, VEL 30, DEL 150, PUL 80;

Line 2: HEAT 650, FIL 3, VEL 40, DEL 135, PUL 150.

S2. Illustration of one completed SECCM measurement

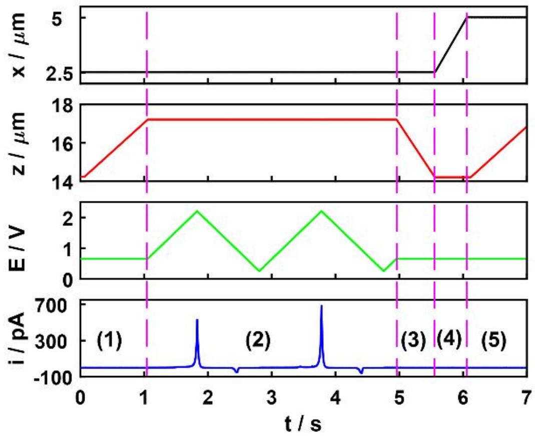

Fig. S1 Plots of variation of four parameters (x-piezo movement, z-piezo movement, potential output of potentiostat, activity current response) with time, divided into five separate processes, show one completed measurement at one spot: (1) approach and meniscus contact, (2) two-cycle CV measurement at one spot, (3) retract of pipette, (4) movement to x-piezo to next spot, (5) approach to initiate next measurement. Note that the applied voltage (green) is shown versus RHE.

S3. Additional macroscale cyclic voltammetry on gold

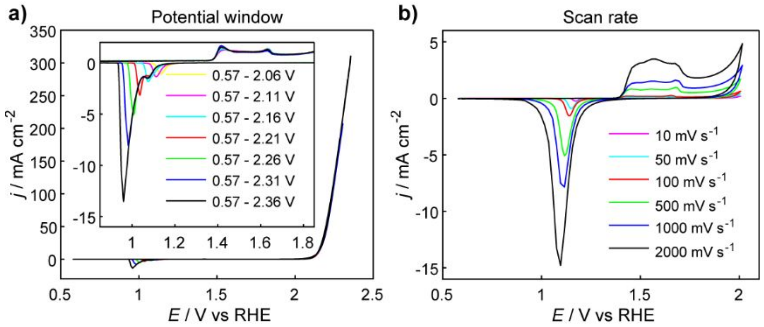

Fig. S2 Cyclic voltammograms obtained at the macroscale with a) different anodic potential limits and b) scan rate (anodic potential limit 2.36 V), using a polycrystalline gold nugget as the working electrode in the standard 3-electrode configuration. For the inset image on a) the anodic part was magnified by ten times in the potential window of 0.8 - 1.85 V.

Table S1 The value of charges on the reduction of gold oxides and current at 2 V

Scan Rate / mV s^{-1}	Reduction charges /μC	
	1.1-1.5V	0.6-1.5V
10	60.02	56.35
50	58.89	62.57
100	54.77	59.94
500	39.90	51.35
1000	30.36	46.48
2000	24.22	48.68

S4. Movie captions

Movie S1. SECCM electrochemical movie includes 920 independent cyclic voltammetry measurements on the surface of gold nugget, with a pixel density of 0.16 pixel μm^{-2}. The voltammetric measurement was performed at a scan rate of 2 V/s, with the potential range from 0.29 V to 2.24 V vs. RHE.

Movie S2. SECCM electrochemical movie includes 1640 independent cyclic voltammetry measurements on the surface of gold nugget, with a pixel density of 0.16 pixel μm^{-2}. The voltammetric measurement was performed at a scan rate of 2 V/s, with the potential range from 0.29 V to 2.24 V vs. RHE. The detailed analysis is presented in **Fig. S5** and **Table S4**.

Movie S3. SECCM electrochemical movie includes 1600 independent cyclic voltammetry measurements on the surface of gold nugget, with a pixel density of 0.16 pixel μm^{-2}. The voltammetric measurement was performed at a scan rate of 2 V/s, with the potential range from 0.29 V to 2.24 V vs. RHE. The detailed analysis is presented in **Fig. S6** and **Table S5**.

Movie S4. SECCM electrochemical movie includes 1209 independent cyclic voltammetry measurements on the surface of gold nugget, with a pixel density of 0.16 pixel μm^{-2}. The voltammetric measurement was performed at a scan rate of 2 V/s, with the potential range from 0.28 V to 2.23 V vs. RHE. The detailed analysis is presented in **Fig. S7** and **Table S6**.

Movie S5. HR SECCM electrochemical movie includes 378 independent cyclic voltammetry measurements on the surface of gold nugget, with a pixel density of 156.25 pixel μm^{-2}. The voltammetric measurement was performed at a scan rate of 2 V/s, with the potential range from 0.29 V to 2.19 V vs. RHE.

Movie S6. HR SECCM electrochemical movie includes 150 independent cyclic voltammetry measurements on the surface of gold nugget, with a pixel density of 156.25 pixel μm^{-2}. The voltammetric measurement was performed at a scan rate of 2 V/s, with the potential range from 0.29 V to 2.14 V vs. RHE.

S5. Additional analysis of SECCM measurement in Fig. 3

S5.1 The current density distribution extracted from SECCM measurements

Table S2 The current density distribution of four grains shown in Fig. 3

Grain		A	B	C	D
Pixels		20	412	365	107
Euler angles	Φ_1	339.81	75.64	118.37	47.07
	Φ	22.86	41.34	13.37	51.35
	Φ_2	54.98	4.86	35.61	47.65
	Index	<113>	<011>	<114>	<111>
Current density (Mean±SD) / mA·cm^{-2}	1.54 V	12.1±0.5	15.3±0.7	10.9±0.4	6.6±0.4
	1.00 V	-99.2±2.4	-70.0±2.4	-93.0±2.5	-80.4±1.8
	2.13 V	59.7±2.0	62.5±2.2	60.5±1.6	60.4±1.7
	2.19 V	213.1±3.1	221.6±8.3	225.8±5.5	226.2±3.8
	2.23 V	752.6±15.4	798.6±32.3	808.4±19.8	826.9±27.0

S5.2 Comparison of current density of different grains

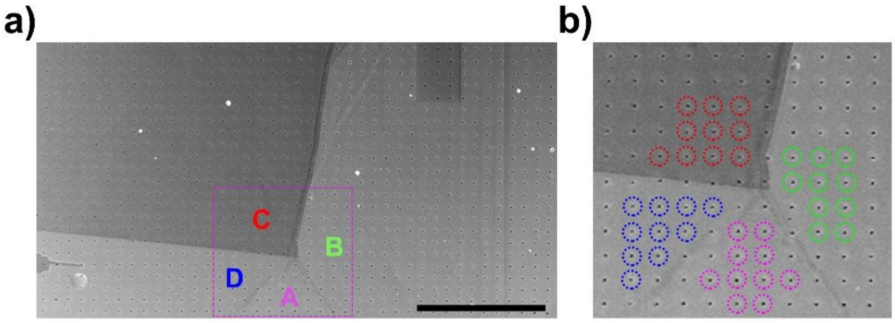

Fig. S3 a) SEM image of the surface of polycrystalline gold nugget, acquired after SECCM experiment. The scale bar is 25 μm. The magenta marked square area was magnified and shown in b), where ten pixels marked with magenta, green, red and blue cycles, corresponding to the grain A, B, C and D in Fig.3, were selected for detailed analysis.

To minimize the effect of potential drift of QRCEs on the current densities in larger grains, ten pixels of measurements from each grain, which are spatially close to each other and not covering the grain boundaries, are averaged to represent the current density (M_{CD}). Then the average current densities at 2.14V and 2.20 V are used to compare the increasing rate within each grain. And the average current densities at 2.20 V are used to compare the discrepancy between each grain. Take the grain A and D of Fig. 3 as an example:

$$Increasing\ rate = \frac{M_{CD\_2.20V} - M_{CD\_2.14V}}{(2.20 - 2.14) \times 1000}$$

$$Ratio = \frac{M_{CD\_Grain\_D} - M_{CD\_Grain\_A}}{M_{CD\_Grain\_A}}$$

where $M_{CD\_2.20V}$ and $M_{CD\_2.14V}$ are the averaged current density of one grain at the voltage of 2.20 and 2.14 V, respectively. The $M_{CD\_Grain\_D}$ and $M_{CD\_Grain\_A}$ represent the averaged current density of grain D and grain A at the voltage of 2.20 V.

S5.3 Illustration of oxidation charges of various potential ranges

Table S3 The charge of gold oxidation process and relative changes within various grains

Grain	Charge (Q, Mean±SD) / pC					
Potential	1.2-1.54 V	1.2-1.85 V	1.2-2.0 V	1.2-2.13 V	1.2-2.19 V	1.2-2.23 V
A	0.53 ± 0.02	1.46 ± 0.03	2.04 ± 0.04	3.54 ± 0.05	6.51 ± 0.05	13.83 ± 0.15
B	0.36 ± 0 01	1.30 ± 0.03	1.86 ± 0.03	3.44 ± 0.04	6.55 ± 0.08	14.30 ± 0.30
C	0.53 ± 0.01	1.36 ± 0.02	1.91 ± 0.03	3.39 ± 0.04	6.46 ± 0.06	14.24 ± 0.20
D	0.21 ± 0.05	1.35 ± 0.04	1.85 ± 0.05	3.30 ± 0.07	6.40 ± 0.08	14.30 ± 0.16
Relative changes	$(Q_{max}-Q_{min})/Q_{max}$					
	0.61	0.11	0.09	0.07	0.02	0.03

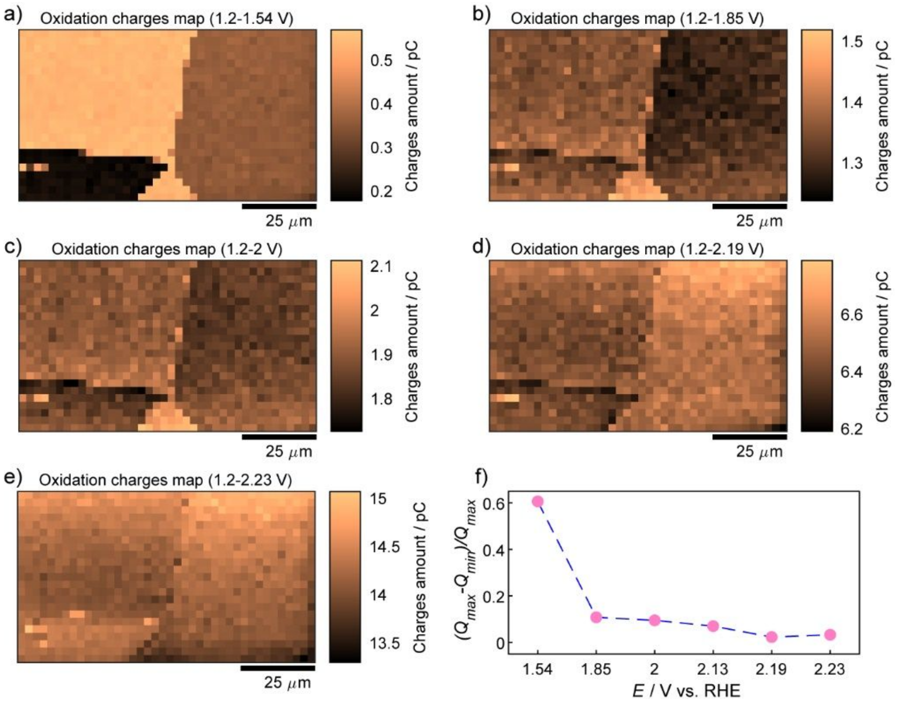

Fig. S4 The oxidation charges map, built by integrating the anodic CV curves of each pixel in the potential window from 1.2 V to a) 1.54V, b) 1.85 V, c) 2.0 V, d) 2.19 V and e) 2.13 V, respectively. f) The relative changes of gold oxidation charges between four grains under various potential window. The x-axis represents the upper potential limit of various potential windows corresponding to Fig. 4b and Fig. S4a-S4e.

S6. Additional SECCM measurements on gold surface

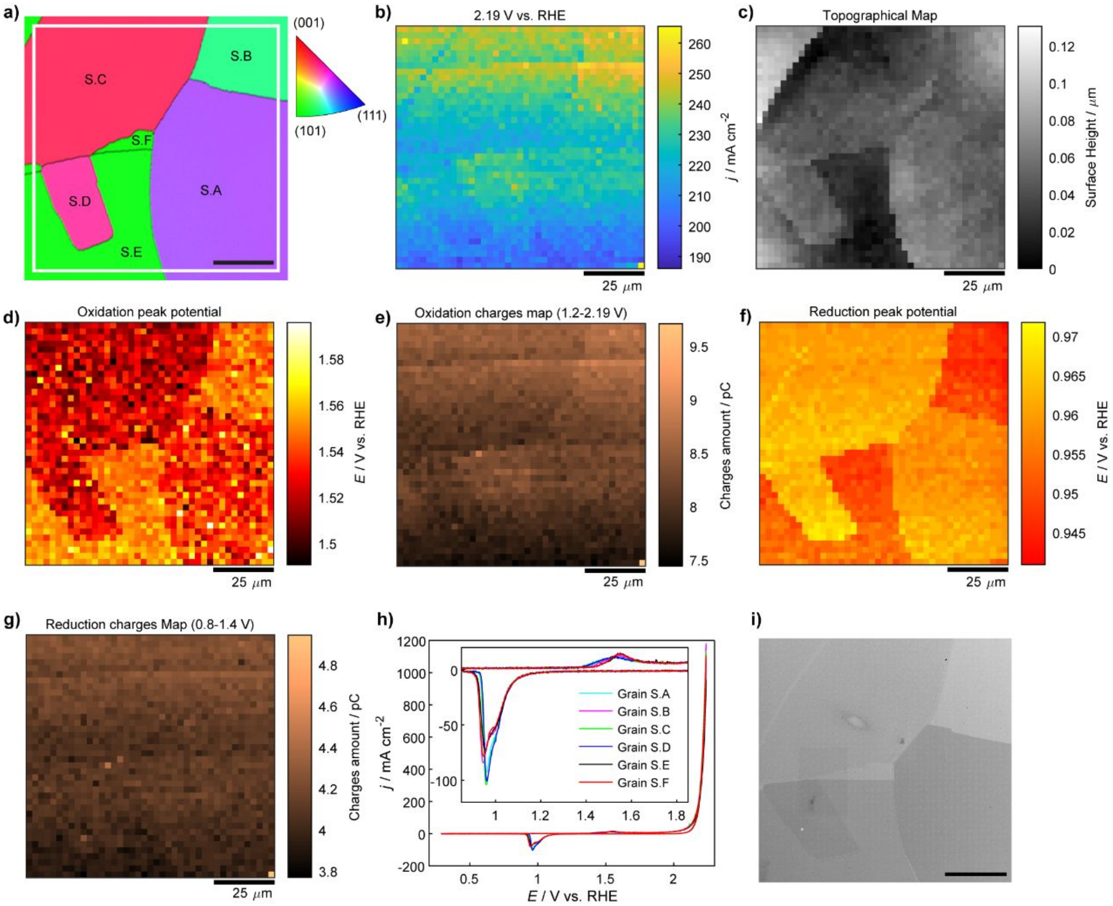

Fig. S5 a) The EBSD grain orientation map of the surface of a polycrystalline gold nugget, acquired after the SECCM experiment. The scale bar is 25 µm. Spatially-resolved equipotential snapshot image got from Movie S2 at the specific potential of b) 2.19 V. c) The topographical map of the scanned area from the SECCM scan. d) The oxidation potential shift map, built by finding the main oxidation peak potential of anodic CV curves of each pixel. e) The oxidation charges map, built by integrating the anodic CV curves of each pixel in the potential window of 1.2 - 2.19 V. f) The reduction potential shift map, built by finding the main reduction peak potential of cathodic CV curves of each pixel. g) The reduction charges map, built by integrating the cathodic CV curves of each pixel in the potential window of 0.8 - 1. 4 V, which covers all the reduction peak region. h) The representative cyclic voltammogram curves of six grains obtained from the SECCM measurement. The inset images are magnified cyclic voltammogram curves in the potential window of 0.8 – 1.85 V. i) SEM image of the surface of the polycrystalline gold nugget, acquired after the SECCM experiment. The scale bar is 25 µm.

Table S4 The current density distribution of six grains shown in Fig. S5

Grain	Pixels	Euler angles				Current density (Mean±SD) / mA·cm^{-2}		
		Φ_1	Φ	Φ_2	Index	1.46 V	0.98 V	2.19 V
S.A	440	262.88	32.26	56.28	<214>	7.16±0.51	-70.31±2.96	218.85±10.00
S.B	172	349.53	40.58	18.59	<134>	5.91±0.47	-58.01±1.90	242.42±7.56
S.C	615	181.9	16.89	31.98	<114>	8.10±0.45	-78.08±2.86	232.01±9.75
S.D	111	249.49	22.48	34.94	<113>	7.60±0.42	-79.55±3.98	215.47±7.79
S.E	267	153.45	41.09	85.7	<101>	4.05±0.44	-56.98±2.18	215.77±11.36
S.F	24	76.54	41.21	4.45	<011>	4.30±0.42	-55.91±1.78	231.77±4.63

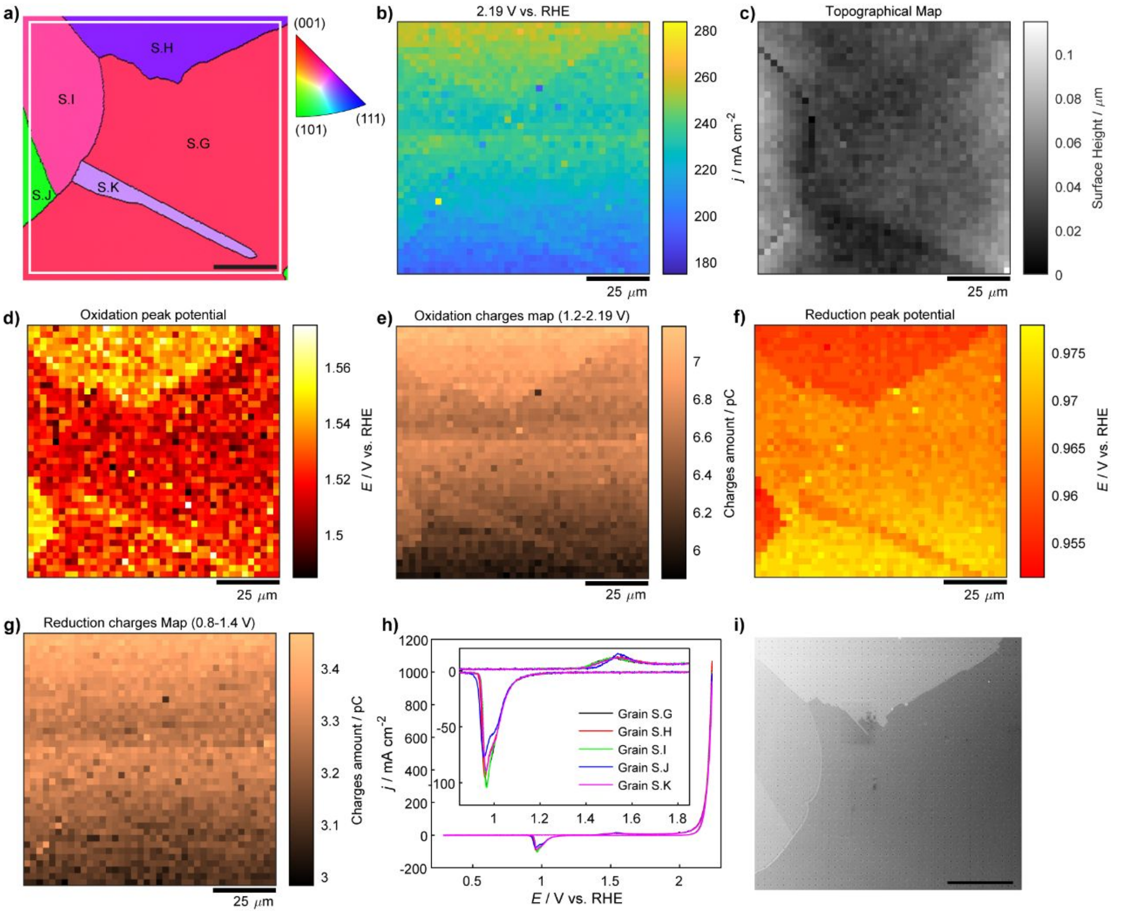

Fig. S6 a) The EBSD grain orientation map of the surface of polycrystalline gold nugget, acquired after SECCM experiment. The scale bar is 25 μm. Spatially-resolved equipotential snapshot image got from Movie S3 at the specific potential of b) 2.19 V. c) The topographical map of the scanned area from the SECCM scan. d) The oxidation potential shift map, built by finding the main oxidation peak potential of anodic CV curves of each pixel. e) The oxidation charges map, built by integrating the anodic CV curves of each pixel in the potential window of 1.2 - 2.19 V. f) The reduction potential shift map, built by finding the main reduction peak potential of cathodic CV curves of each pixel. g) The reduction charges map, built by integrating the cathodic CV curves of each pixel in the potential window of 0.8 - 1. 4 V, which covers all the reduction peak region. h) The representative cyclic voltammogram curves of five grains obtained from the SECCM measurement. The inset images are magnified cyclic voltammogram curves in the potential window of 0.8 – 1.85 V. i) SEM image of the surface of the polycrystalline gold nugget, acquired after the SECCM experiment. The scale bar is 25 μm.

Table S5 The current density distribution of five grains shown in Fig. S6

Grain	Pixels	Euler angles				Current density (Mean±SD) / mA·cm^{-2}		
		Φ_1	Φ	Φ_2	Index	1.57 V	0.97 V	2.19 V
S.G	1003	181.9	16.89	31.98	<114>	9.54±0.52	-97.08±4.23	222.64±14.80
S.H	309	318.96	34.88	41.97	<112>	11.02±0.42	-82.11±2.08	248.66±85.22
S.I	175	250.29	22.14	33.76	<114>	9.46±0.52	-98.27±2.93	237.21±39.26
S.J	37	153.19	40.85	85.89	<304>	12.88±0.82	-69.54±3.47	222.79±10.03
S.K	62	9.51	31.71	60.48	<214>	10.48±0.47	-85.71±2.33	215.30±7.77

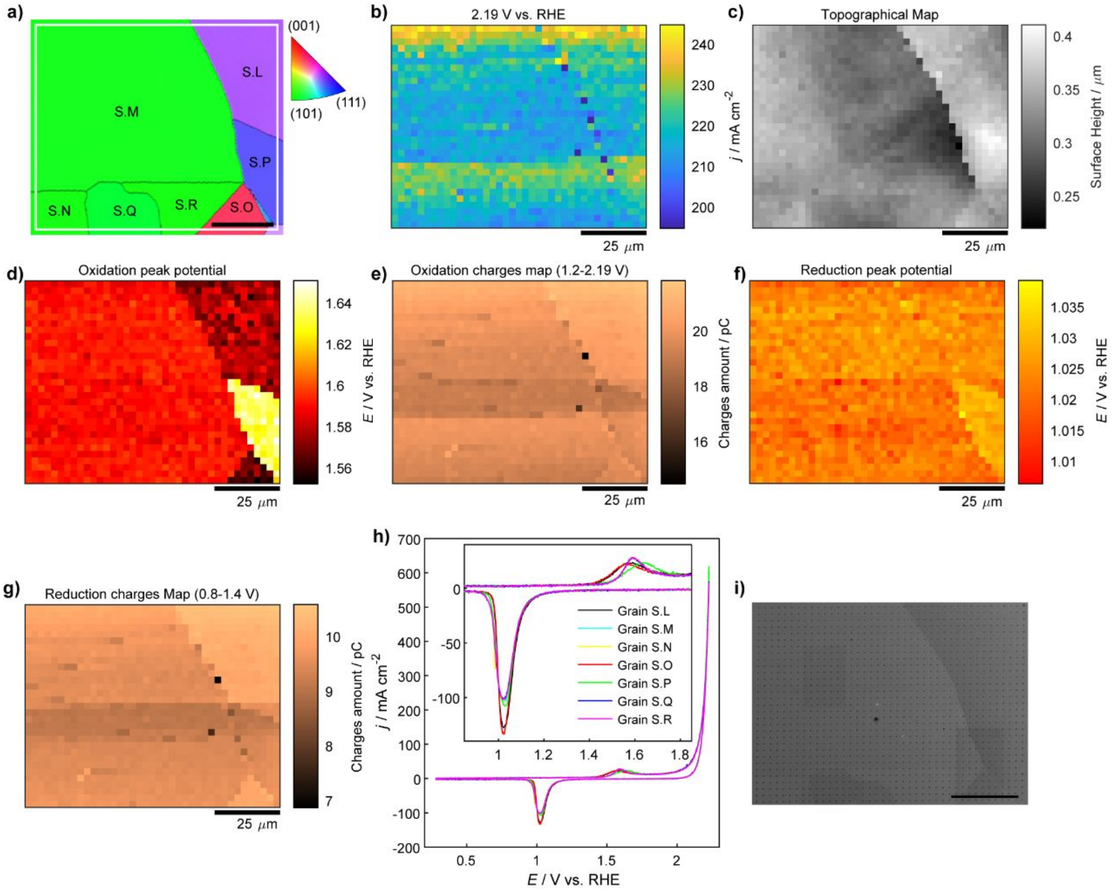

Fig. S7 a) The EBSD grain orientation map of the surface of a polycrystalline gold nugget, acquired after the SECCM experiment. The scale bar is 25 μm. Spatially-resolved equipotential snapshot images got from Movie S4 at the specific potential of b) 2.19 V. c) The topographical map of the scanned area from the SECCM scan. d) The oxidation potential shift map, built by finding the main oxidation peak potential of anodic CV curves of each pixel. e) The oxidation charges map, built by integrating the anodic CV curves of each pixel in the potential window of 1.2 - 2.19 V. f) The reduction potential shift map, built by finding the main reduction peak potential of cathodic CV curves of each pixel. g) The reduction charges map, built by integrating the cathodic CV curves of each pixel in the potential window of 0.8 - 1. 4 V, which covers all the reduction peak region. h) The representative cyclic voltammogram curves of seven grains obtained from the SECCM measurement. The inset images are zoomed-in cyclic voltammogram curves in the potential window of 0.8 – 1.85 V. i) SEM image of the surface of the polycrystalline gold nugget, acquired after the SECCM experiment. The scale bar is 25 μm.

Table S6 The current density distribution of seven grains shown in Fig. S7

Grain	Pixels	Euler angles				Current density (Mean±SD) / $mA \cdot cm^{-2}$		
		Φ_1	Φ	Φ_2	Index	1.55 V	0.97 V	2.19 V
S.L	187	262.88	32.26	56.28	<214>	21.34±0.59	-8.08±0.64	223.77±7.88
S.M	729	153.45	41.09	85.7	<101>	17.36±0.78	-20.36±2.96	220.01±8.61
S.N	36	76.21	41.23	4.74	<034>	16.77±0.52	-21.24±0.63	216.85±3.67
S.O	21	182.42	16.91	31.35	<114>	21.65±0.36	-6.94±0.52	214.88±4.04
S.P	62	99.42	38.92	36.08	<234>	12.62±0.56	-15.78±1.62	221.16±7.93
S.Q	57	194.43	43.52	82.62	<414>	16.92±0.81	-21.60±0.90	215.77±5.12
S.R	87	76.17	41.02	4.65	<034>	16.69±0.51	-21.57±1.04	216.28±4.73

S7. SEM image of footprints after SECCM experiments

In order to rule out the possibility of electrowetting during the SECCM scan, a series of SECCM measurements were performed with different upper potential limits, which were located at three different regions: double layer region, gold oxidation region, and OER region. It is found that the size of all the footprints remains consistent around 500 nm, regardless of the upper potential limit.

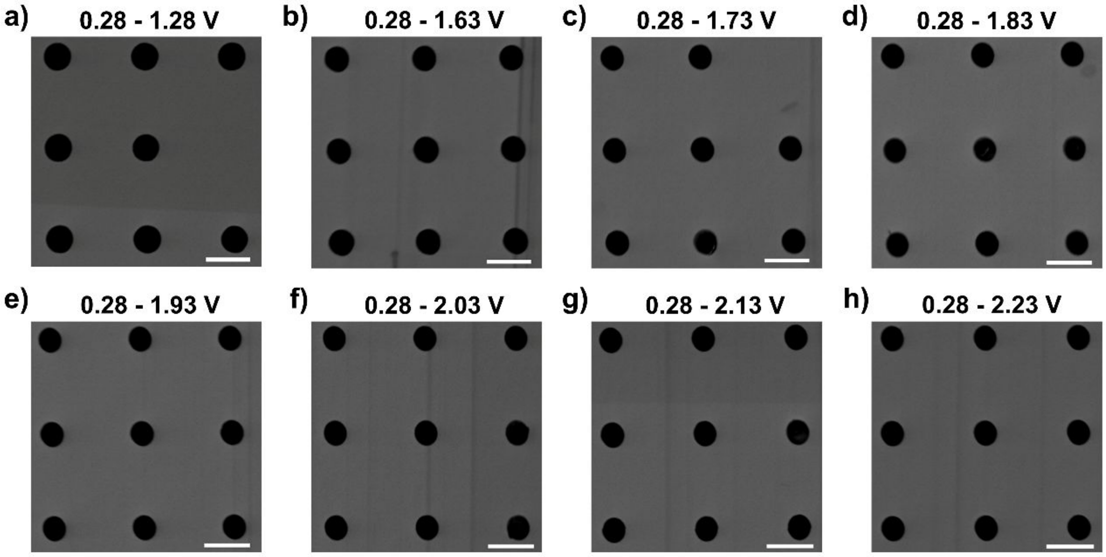

Fig. S8 The SEM images of footprints after SECCM measurements with different upper potential limits. The upper potential of a) is in a double-layer region where no gold oxidation happens. The upper potentials of b) to f) are in the gold oxidation region, where b-d) surface gold oxide film forms and e-f) oxygen partially evolves from surface gold oxides. The upper potential of g) and h) are in the OER region, where oxygen evolves from the electrolyte. The scale bars are 1 μm.

S8. Additional analysis of HR-SECCM measurement in Fig. 5 and Fig. 6

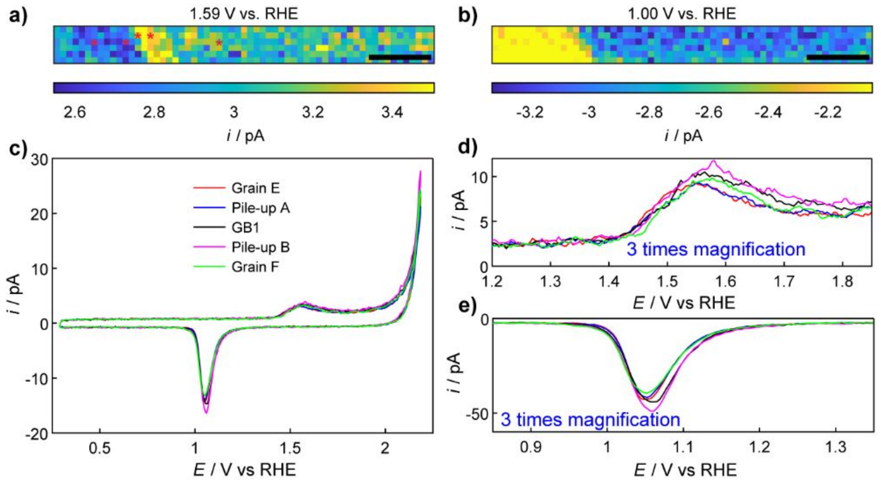

Fig. S9 Spatially-resolved equipotential snapshot images obtained from Movie S5 at specific potentials of a) 1.59 and b) 1.00 V, respectively. The scale bars of a) and b) are 0.8 µm. c) The representative CV curves of grain E, Grain F, pile-up A, pile-up B and corresponding grain boundary GB1. The three times magnified CV curves indicate the grain-dependent d) gold oxidation and e) gold oxides reduction transients. The red stars indicate the position of the representative CVs taken from.

Table S7 The current density distribution of grains and grain boundary shown in Fig. 5

Grain	Pixels	Euler angles				Current/ pA		
		Φ_1	Φ	Φ_2	Index	1.59 V	1.00 V	2.18 V
E	64	326.10	18.63	28.65	<114>	2.84±0.16	-1.90±0.18	19.98±0.64
Pile-up A	15	---	---	---	---	2.76±0.12	-1.96±0.18	18.42±0.49
GB1	10	---	---	---	---	3.32±0.19	-2.26±0.19	20.30±0.84
Pile-up B	22	---	---	---	---	3.42±0.15	-2.80±0.28	22.41±0.62
F	267	337.70	38.54	72.69	<314>	3.08±0.17	-2.94±0.21	20.80±0.84

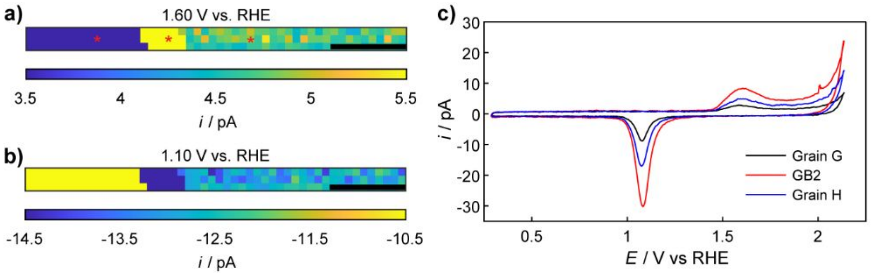

Fig. S10 Spatially-resolved equipotential snapshot images obtained from Movie S6 at specific potentials of a) 1.60 and b) 1.10 V, respectively. The scale bars of a) and b) are 0.8 μm. c) The representative CV curves of grain G, Grain H and corresponding grain boundary GB2. The red stars indicate the position of the representative CVs taken from.

Table S8 The current density distribution of grains and grain boundary shown in Fig. 6

Grain	Pixels	Euler angles				Current / pA		
		Φ_1	Φ	Φ_2	Index	1.60 V	1.10 V	2.13 V
G	45	353.15	42.59	32.7	<234>	2.64±0.16	-6.22±0.53	6.14±0.37
GB2	13	---	---	---	---	7.86±0.81	-25.77±3.11	19.54±1.91
H	87	88.81	23.91	7.98	<012>	4.69±0.20	-12.86±0.54	11.76±0.81

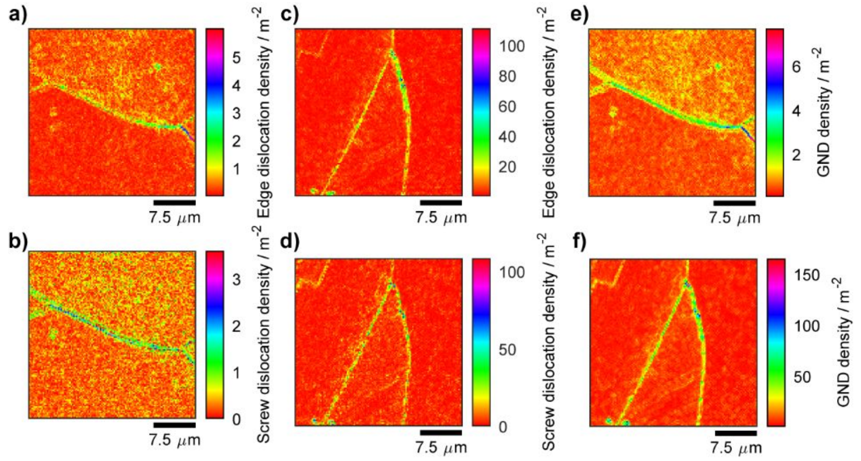

Fig. S11 a) The edge dislocation and b) screw dislocation maps of region Ⅰ. c) The edge dislocation and d) screw dislocation maps of region Ⅱ. The geometrically necessary dislocation (GND) density maps of e) region Ⅰ and f) region Ⅱ.
